# Association of Pain During the Evaluation of Delirium in Intensive Care Unit Patients

**DOI:** 10.3389/fmed.2021.722001

**Published:** 2021-08-24

**Authors:** Evelyn A. Álvarez, Francisco J. Parada

**Affiliations:** ^1^Centro de Estudios en Neurociencia Humana y Neuropsicología, Facultad de Psicología, Universidad Diego Portales, Santiago, Chile; ^2^Escuela de Psicología y Terapia Ocupacional, Facultad de Ciencias de la Salud, Universidad Central de Chile, Santiago, Chile

**Keywords:** pain, intensive care unit, delirium screening, connectome, electroencephalography

## Introduction

The present article aims to discuss how pain can affect cognitive performance, focusing our attention on the evaluation of delirium in critical patients and its relation with pain. To achieve this, we will briefly review (i) neural changes and dynamic adaptation processes between pain and attention; (ii) the effects of acute pain in performance and neural activity of attentional tasks; (iii) the implementation process of delirium's evaluation in hospitalized patients with pain at an intensive care unit. We finally discuss these elements in the light of implementing cognitive assessments processes in pain patients.

## Pain and Attention

We understand pain as an unpleasant sensory, psychological, and emotional experience emerging from the interaction between an agent and a situation associated with threat and potential physical and/or psychological damage, facilitating behaviors oriented toward protecting from such damage. Pain is generally caused by a noxious stimulus triggering physiological processes called nociception, which is mediated by a series of sensory and contextual events (behavioral, emotional, cognitive, and sociocultural). Thus, pain is a dynamic bodily-effective integrative phenomenon (1). Given its high dimensionality, it could be said that pain demands a high level of cognitive resources, interfering, distracting, and demanding the focusing of attention (2) and thus decreasing the offloading and extending capacities of cognition (3, 4). Depending on the temporality of a painful experience, it can be classified as acute or chronic. Acute pain occurs in the short term and can be resolved within 3 to 6 months, being the response to an injury or specific trauma and acting as a warning system for the body. When tissue damage is involved, this type of pain is self-limited and it is resolved with cicatrization. In some cases, acute pain can turn into chronic pain, which we understand as intermittent or persistent pain experienced during more than 3 months and/or persisting beyond healing time (5). Chronic pain has been observed to affect attention in demanding tasks (6). In healthy individuals, any type of pain causes immediate protective responses, such as safety-seeking behaviors, including escape and avoidance. Attention protects the pursuit of current goals by inhibiting the processing of irrelevant information (6).

### Neural Changes and Dynamic Processes Between Pain and Attention

At a neural processes level, pain has been assessed through functional/structural connectivity (i.e., connectomics) and cognitive electrophysiology using electroencephalography (EEG). Connectomics allows us to understand the connectivity and communication among brain networks, analyzing the nodes and functional and anatomic tracts interconnections through complex-network analysis (7). Relevant to our case is the identification of the salience network, which comprises brain areas such as the anterior insula, medial cingulate cortex, temporoparietal junction and prefrontal cortex. The intrinsic activity or resting-state network composed by posterior cingulate cortex, medial prefrontal cortex, lateral parietal lobe, along with medial temporal lobe areas has also been shown as relevant in the process of pain detection in conjunction with an antinociceptive modulation system that includes the central region of the periaqueductal gray area in the brain stem (7). Thus, when attention is directed toward a painful stimulus, the medial prefrontal cortex exercises causal influences over the primary sensorimotor cortex. In contrast, by removing attention (i.e., distraction), this influence disappears (1). The foregoing suggests dynamism from the information flow's context in pain processing, being an adaptable network with functional connections, neurobiologically constructing the painful experience as a flexible phenomenon (8).

Furthermore, electrophysiological evidence shows that neural oscillatory activity -described in terms of frequency, phase and amplitude- codifies specific activity (9). EEG dynamics allow quantifying the brain's functional integration and segregation processes. Low-frequency oscillations (<30 Hz) can be found at the base of the signal known as event-related potentials (ERP), which are an average of neural activity at time-domain (Box 1) (19). ERP studies related to pain, where stimuli are caused by nociceptive laser stimuli, show modulations in the complex known as N2-P2, occurring within the 200–400 ms latency range when the back of the hand is stimulated (20). Other studies have shown that pain stimuli generate above-threshold voltage deflections in the N1-N2 components, in contralateral somatosensory areas or in the posterior insula. Considering neural activity in time and frequency domains, broadband and frequency specific changes in Theta (4–8 Hz), Alpha (8–15 Hz), Beta (15–30 Hz), and Gamma (>30 Hz) oscillations have been identified in the nociceptive processing during the presence of chronic and acute pain (Box 1). Furthermore, functional coupling of Theta and Gamma involves nodes in both the salience and default network in chronic pain (21).

Box 1 **Neural activity's information description through EEG.**

**Event-related potentials (ERP)**.

The ERP have an adequate temporal resolution that allows the measurement of the brain activity relevant to attentional and perceptual tasks (10). The electric responses from the cortex generate voltage fluctuations, which are measured by the EEG. These fluctuations are temporally related to motor or cognitive sensory events. In general, these are generated as a response to peripheral or external stimulations and appear as somatosensory, visual and hearing brain potentials, or as a slow-evolution brain activity observed before voluntary movements or during the anticipation of the conditional stimulation (11).

That is how attentional and pain aspects related to ERP have been found, such as:

- N1 with spatial attention and discrimination tasks, along with pairing processes (12)
- P2 with the sensation-seeking behavior (13)
- N2 with relevant and disparate stimuli identification
- LPP with emotional intensity stimuli (14)
- P300 obtained from parietal and central areas as a response to visual or hearing salient stimuli (15)

**Frequency bands**

Brain oscillations recorded by EEG have been classified into frequency bands, and through research work, they have been associated with different brain states or functions (16). A brief description of them are listed below:

- Delta waves (up to 4 Hz) are high-amplitude waves that have been associated with deep sleep stages, in awake subjects with cortical plasticity, as a participating oscillation in cognitive processes of event-related potentials, contributing within P3, and also associated with the assessment of stimuli and the decision-making process (17). Detection of pain (6).
- Theta wave (4–8 Hz). On a frontal level, it has a role within attention processing, cognitive control and working memory (18)
- Alpha wave (8–13 Hz) manifests itself spontaneously in adults during the vigil and in a relaxed state, and shows variations during working memory retention. Sensitivity to pain
- Beta wave (13–25 Hz). It has been observed greater activity in the frontal and central areas during the execution of activities, resolution of problems and sustained attention. During spatial discrimination tasks and visual attention, activity in occipital areas has been observed. It detects sensory variations and pain
- Gamma waves (over 25 Hz) are quick oscillations that can be found during conscious perception. It is also related to attentional processes, working memory and long-term memory (14). Pain saliency (8, 16).

Evidence suggests that when cognitive, behavioral, and contextual resources are required, attention can optimize resources toward goal-relevant information (22). Pain -as an inherent prominent stimulus (23)- and nociceptive processing are closely intertwined with attentional processes (8), situating and embedding the dynamic integration of sensory and contextual processes during painful experiences (1).

### Pain-Related Neural Dynamics During Attentional Tasks

In a meta-analysis conducted in 2019 (6), eight studies describing the influence of acute pain and attentional changes were found, considering the following subcomponents: (i) *orientation*, five studies evaluated spatial signals, where they found that subjects with pain presented more errors in the identification of non-valid signals; (ii) *awareness*, two studies conducted continuous performance assessments and found that pain diminishes alertness; and (iii) *executive attention*, two studies used Go/No-Go and Stroop paradigms to detect slight changes in response time and performance. Considering this evidence, pain changes both the performance and execution of attentional tasks. Similarly, a recently published quasi-experimental study measured pain-modulated neural activity during attentional tasks (24). Participants performed an executive attention task, in which the subjects had to remember a series of numbers in two conditions: low-cognitive load (i.e., two digits) or high-cognitive load (i.e., six digits). The experimental group reported moderate pain after capsaicin-induced painful stimulation. Neural activity was recorded using EEG. Results show that the experimental group decreased their behavioral accuracy without supra-threshold ERP differences considering cognitive load. In contrast, the control group reported above-threshold ERP differences between low- and high-load cognitive tasks. Authors interpret their results as indicating participants experiencing pain decrease attention to cognitive demands, affecting the use of appropriate strategies to solve the task, allocating similar attentional resources in cognitive tasks with different load. Furthermore, frequency analysis showed that Delta and Theta oscillations exhibit a delay in high-load tasks for the subjects within the experimental group considering cognitive load. This study showed that pain and cognitive load affect both cognition and behavior, interfering in the accuracy of the responses. Furthermore modulating early stages of attention, impeding participants experiencing pain to adjust to task demands both at behavioral and neurophysiological levels. Thus, the experience of acute nociceptive stimulation has significant changes in attention with relevant neurobehavioral consequences.

## Pain and Attention at the Intensive Care Unit

Pain is a permanent concern and a common symptom among critically ill adults. Approximately 50-80% of the patients report pain at rest and/or during procedures (25) caused by the process of primary diseases and tissue injury, invasive procedures, endotracheal suction, presence of endotracheal and nasogastric tubes, invasive monitoring catheters, urinary catheters, drainage, insertion and extraction of tubes and catheters, wounds care, immobility and position changing. These medical procedures are described by patients as painful interventions (26–28). It is important to emphasize that patients describe their pain as a significant part of their intensive care unit (ICU) experiences (29), classifying it from moderate to severe (28). Considering that pain is complex and subjective, self-report is still the gold standard for its detection and evaluation. Given that ICU patients are critically ill, intubated, and with different levels of sedation, pain detection is a permanent challenge because communication and self-report opportunities are often limited (27). Furthermore, ICU patients are prone to developing delirium. Delirium is a neuropsychiatric disorder that occurs in an acute and fluctuant manner, affecting attention and other cognitive areas (30). It generates long-term complications [i.e., longer periods of mechanical ventilation (31), hospital stay (32), cognitive and mental health impairment (33, 34), caretakers' overload (35), mortality (36)].

Considering the effects of pain, the implementation of timely monitoring becomes relevant. Thus, several guidelines (37, 38) suggest the use of instruments, the most well-known being (i) Confusion Assessment Method (CAM-ICU) and (ii) Intensive Care Delirium Screening Checklist (ICDSC) (39). These assessment methods are based on brief interviews and simple tests that measure cognitive function focusing on attention (i.e., instruction following, alertness, sustained attention). These instruments take into account the use of sedation scales measuring the state of consciousness in a quantitative manner. Nevertheless, they neglect pain evaluations, which can directly interfere in orientation, awareness, and executive attention.

Finally, ICU patients' condition who are constantly exposed to pain is highly complex due to the challenging non-ecologic physical environment in which they are embedded (i.e., environmental noise, regulation of the sleep-wake cycle, temperature, artificial light, etc.) and their physical/volitional restrictions (i.e., equipment for drug's administration, bladder-bowel control management, respiratory control, etc.) (40) is prone to generate overloaded nociceptive stimulation and experiences. Hence, researchers and practitioners within the ICU environment must ask ourselves, what is the proper form of an ICU delirium evaluation? How can it be properly implemented in this type of patient? and How to interpret the information obtained from such an assessment?. We are ways away to answer these and other questions.

## Concluding Remarks

In the present piece, we briefly reviewed the current evidence showing how pain impacts cognitive functioning, specifically attention. Furthermore, we conceptualize pain as a dynamic and flexible process that generates salience effects linked to specific neurodynamics in large thalamocortical networks related to perception, cognition, and behavior. Laboratory-based evidence can provide us some insights to understanding people's neurobehavioral states when in fragile and high nociceptive demanding situations. These can be thought of as a model for ICU patients, where pain is reported as a frequent complication and where their cognitive skills show an apparent performance deterioration. More specifically, attentional and thought-organization abilities measured with delirium assessments, appear as heavily compromised. Thus, we invite delirium researchers and practitioners to consider pain -in its complexity- as a main factor during delirium evaluations.

Furthermore, since assessing delirium depends on evaluating attentional and thought-related alterations, identifying and understanding the experience of pain can be a key component toward improving our practices. Thus, the main goal of the present piece is to invite researchers and practitioners to discuss delirium assessment practices and procedures to improve its implementations and interpretation of results, especially in the ICU context which facilitates painful experiences. Hence, understanding the unique coupling parameters and constraints enabling and/or constituting patient states embedded in the ICU, will provide better orientation for assessment, treatment or intervention rationale, and a shared language for interdisciplinary dialogue and research integration.

## Author Contributions

EÁ wrote the first manuscript draft. EÁ and FP wrote and edited the final manuscript version for publication. All authors contributed to the article and approved the submitted version.

## Conflict of Interest

The authors declare that the research was conducted in the absence of any commercial or financial relationships that could be construed as a potential conflict of interest.

## Publisher's Note

All claims expressed in this article are solely those of the authors and do not necessarily represent those of their affiliated organizations, or those of the publisher, the editors and the reviewers. Any product that may be evaluated in this article, or claim that may be made by its manufacturer, is not guaranteed or endorsed by the publisher.
